# Celtic Provenance in Traditional Herbal Medicine of Medieval Wales and Classical Antiquity

**DOI:** 10.3389/fphar.2020.00105

**Published:** 2020-02-28

**Authors:** Charles Wagner, Jillian De Gezelle, Slavko Komarnytsky

**Affiliations:** ^1^ Plants for Human Health Institute, North Carolina State University, North Carolina Research Campus, Kannapolis, NC, United States; ^2^ Department of Plant and Microbial Biology, North Carolina State University, Raleigh, NC, United States; ^3^ Department of Food, Bioprocessing, and Nutrition Sciences, North Carolina State University, Raleigh, NC, United States

**Keywords:** medicinal plants, ethnopharmacology, ethnobotany, herbal texts, bioactive compounds

## Abstract

The Celtic linguistic community dominated large spans of Central and Western Europe between 800 BC and 500 AD, but knowledge of their traditional medicine is very limited. Multiple progressive plant gains in Neolithic settlements along the Danube and up the Rhine valleys suggested that taxon diversity of gathered plants peaked at the Balkans and was subsequently reduced as crop and gathered plants packages were adopted and dispersed throughout Neolithic Europe. This process coincided with the Bronze Age migration of the R1b proto-Celtic tribes, and their herbal traditions were occasionally recorded in the classic Greco-Roman texts on herbal medicines. The provenance of Celtic (Gallic) healing methods and magical formulas as recorded by Pliny, Scribonius Largus, and Marcellus Empiricus can still be found in the first part of the medieval Welsh (Cymry) herbal manuscript *Meddygon Myddfai* (recipes 1–188). Although the majority of *Myddfai* I recipes were based on the Mediterranean herbal tradition of Dioscorides and Macer Floridus, they preserved the unique herbal preparation signatures distinct from continental and Anglo-Saxon counterparts in increased use of whey and ashes as vehicles for formulation of herbal remedies. Six plants could be hypothetically attributed to the Celtic (Welsh) herbal tradition including foxglove (*Digitalis purpurea* L.), corn bellflower (*Legousia speculum-veneris* L.), self-heal (*Prunella vulgaris* L.), sharp dock (*Rumex conglomeratus* Murray), water pimpernel (*Samolus valerandi* L.), and river startip (*Scapania undulata* L.) This review provides initial evidence for traces of Celtic framework in the Welsh herbal tradition and warrants further investigations of bioactivity and clinical applications of the described plant leads.

## Introduction

The Celts were an Iron Age European cultural group that originated from a compact Proto-Indo-European linguistic community somewhere in the border region of Eastern Europe and Western Asia, and invaded into Central and Western Europe by land, as it seems on foot, *circa* 3000 BC. In their footsteps, proto-Celts likely followed the Balkan (Danube) route of the Neolithic Anatolian farmers (Hofmanová et al., 2016) and Yamna steppe pastoralists (Haak et al., 2015) to establish two secondary refuges in the Hallstatt region of Central Europe (Smrcka, 2009) and the Tartessos region of the Iberian peninsula (McEvoy et al., 2004). In doing so, they overtook the local Corded Ware groups (*ca*. 2400 BC) and admixed with the late Bell Beaker groups (*ca*. 2200 BC) in Central Europe by forming a succession of Unetice (2300–1600 BC), Tumulus (1600–1200 BC), and Urnfield (1300–750 BC) cultures likely ancestral to the Celtic speakers. Proto-Celts also spread their influence into the British Isles sometime after 800 BC, perhaps in a very similar manner to how the Bell Beaker culture replaced the late Mesolithic populations of Britain (Olalde et al., 2018) and Ireland (Cassidy et al., 2016). While some areas in Europe preserved the original non-Indo-European languages (Basque, Etruscan, Lusitanian, Rhaetian), populations that spanned the Hallstatt to Tartessos corridor adopted Celtic linguistic and cultural traits in connection with expanding trade networks. Today, the term Celtic generally refers to the living languages and respective cultures of Goidelic (Irish and Scottish Gaelic, and Manx Gaelic from the Isle of Man) and Brittonic (Breton, Cornish, and Welsh) groups spoken by the insular Celts of the British Isles and Brittany. The continental Celtic languages were gradually replaced by Vulgar Latin and Germanic languages sometime after 500 AD (Helix, 2010).

Knowledge regarding the history of traditional Celtic medicine is exceedingly uncertain. A small number of inscriptions and place names that survived in the Lepontic (500–300 BC), Gallic (200 BC–400 AD), and Celtiberian (200–100 BC) continental Celtic languages made no references to their medical tradition, while the earliest inscriptions in insular Celtic languages did not appear before 400 AD (Helix, 2010). As such, most of the original accounts of Celtic culture were recorded by foreign writers immediately before and during the period of the Roman Empire. The lack of direct textual or archaeological evidence of medical practice in the Celtic world largely obscured differentiation of local Celtic medical tradition from the later Mediterranean influences. In this review we argue that traces of Celtic herbal knowledge persisted in the wider European medical tradition by means of a direct oral transmission, and albeit at low frequency, were incorporated into classic and medieval herbal compilations.

## Methods

Middle Welsh, Anglo-Saxon, Roman, and Continental European sources were reviewed in their original languages with the aid of lexicons, dictionaries, as well as both antique and modern English translations. Using *Myddfai I* as one of the earliest known medical texts written in a Celtic language, the most pertinent Roman sources were reviewed in chronological order to observe species frequencies and gather facts relatable to *Myddfai I*. Conclusions are original to the authors and when possible corroborated by citations from other scholarly works. The criteria for the selection of antique works to compare plant species frequencies was based upon the lineage and historical popularity of each text, availability, and relevance to medieval Wales (chronology, location). Herbal preparation signatures were evaluated by calculating the frequencies of certain vehicles used to crush, dissolve, or extract plant material in order to discern how different cultures were uniquely using similar plants. Methods, including translation notes, are discussed in more detail where relevant.

PubMed, Scopus, Google Scholar, ScienceDirect, JSTOR, and NCBI were used to find and review relevant modern research articles. The plantlist.org was used for aligning Scientific Binomials. Archaeobotanical literature was reviewed with a focus on samples taken from the contexts (burials, pottery, pit fills, internal occupation deposits, hearths, ash layers, floors, burnt areas, and middens) likely to represent the accumulated debris from a range of intentional plant-related activities, including the processing of gathered plants and cultivated crops.

## The Celts—An Ethnolinguistic Group

A possible date for the integration of tribal groups and enclaves into the Celtic lineage likely coincides with the merger of the ancestral Urnfield farmers with the Tumulus (kurgan) warlords. The survival of this social structure required continuous conquest and expansion into new territories. The evidence for this is seen in the Celtic migrations over much of Europe as far as the Ukrainian Carpathians, and seemingly universal acceptance of Celtic languages as *lingua franca*, the main communication language in the region (Scheeres et al., 2014). The control over salt mines of the Hallstatt region and the key trade routes to the Mediterranean coast, especially Massalia (Marseille), brought a surplus of trade and accumulation of wealth and power in the hands of the few Celtic elites—and ended as a direct result of the successful expansion of the Roman empire.

### Genetic History of Celtic Tribes

Modern European men are classified into seven most frequent but distinct haplogroups based on the SNP (single nucleotide polymorphism) mutations found on the non-recombinant portion of the Y chromosome (Jobling and Tyler-Smith, 2003). Haplogroup F moved out of Africa into the Arabian Peninsula and Near East circa 45000 BC, and produced a succession of haplogroups K > I, N, P > R in Western Central Asia (Soares et al., 2010). The two R1 subclades, R1a and R1b, established themselves in the Pontiac steppes of Ukraine, while the N2 and N3 subclades spread out to Northeastern Europe *ca*. 25000 BC (Haak et al., 2015). Haplogroup F also produced haplogroup I that moved into the Balkans around 20000 BC. Together, these haplogroups survived the Last Glacial Maximum in the Iberian, Balkan, Ukrainian, or Siberian refuges and re-emerged as major Mesolithic hunter-gatherer groups in Europe when climates improved. The other populations arrived to Europe as Neolithic Anatolian farmers that belonged to the G2a, J2, and the African E3b lineages (Kivisild, 2017). The late Neolithic-early Bronze archaeological horizon of Europe (2900–2400 BC) was dominated by the R1a Corded Ware people in the east and the late R1b Bell Beaker culture in the west. Both cultures met and partially overlapped at the Upper Danube region of modern Germany and Austria (Allentoft et al., 2015). As Yamna steppe pastoralists (predominantly R1b) arrived to the same area *via* the Balkan Danube route, they displayed an opposing approach to the existing cultures in the region, which experienced a rapid decline in human activities due to climatic changes 4000–3000 BC (Kolář et al., 2018). While they largely displaced and pushed the Corded Ware people further to the east, they rather admixed and spread their influence over the Bell Beaker people to the west. This would be possible if we assumed that both the late Bell Beaker and Yamna cultures were at least partially related as evident by the common R1b haplogroup, and preserved some ethnic, linguistic, or cultural identity that allowed for a certain degree of integration. If this assumption is correct, it allows the acceptance of the original theory of east to west migration of the R1b people (R1b-V88), followed by their retreat and subsequent expansion from west to east (Iberian refuge), followed by the second east to west migration of Yamna R1b groups (Rb1-L51) to the area (Haak et al., 2015). Preliminary evidence for a direct genetic R1b link between Yamna and late East Beaker cultures was recently described from Alsace, France (Brunel, 2018).

**Figure 1:**
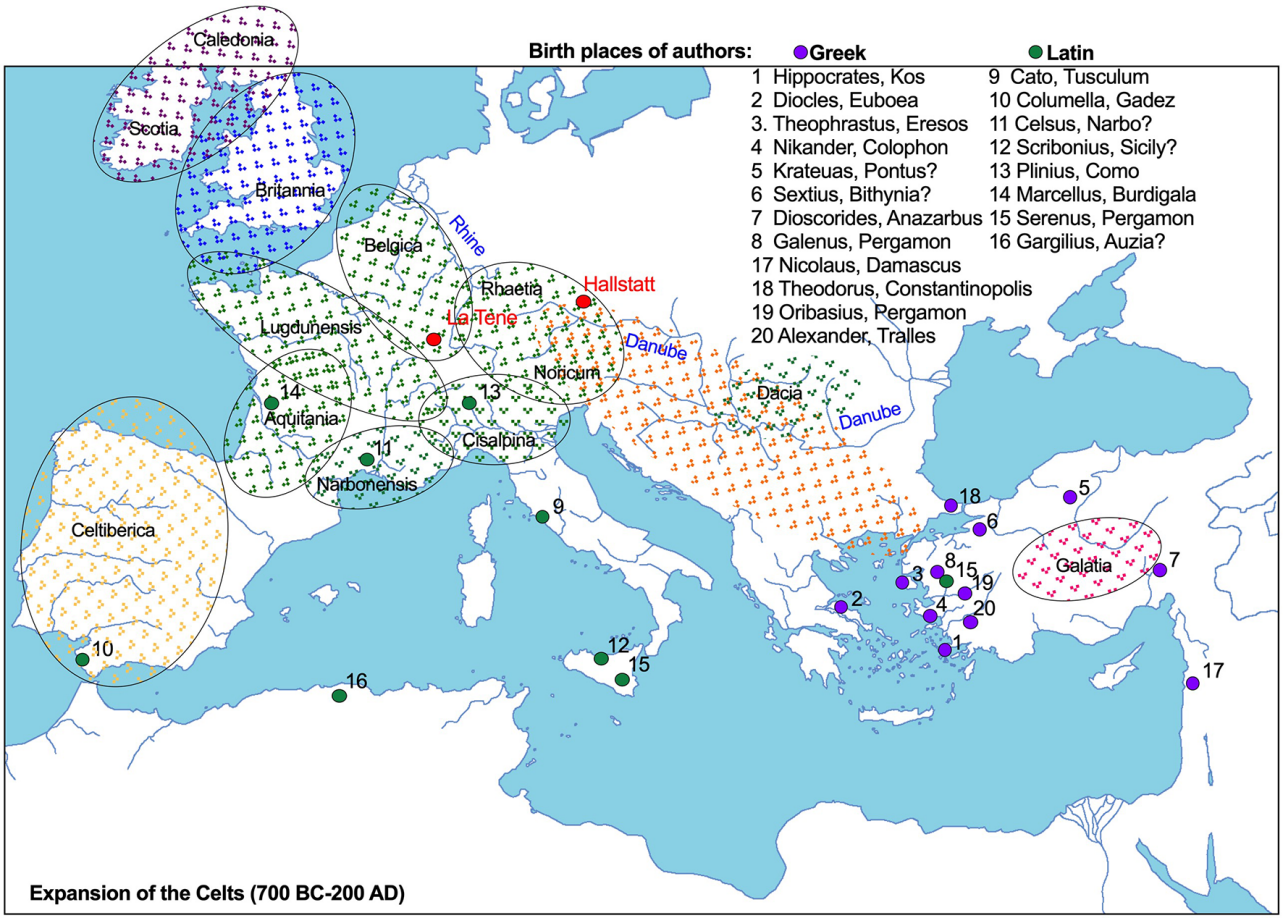
The birth places of Classical medical authors (1–20) who recorded and further developed the use of traditional herbal medicines, in relation to the known Celtic world (700 BC–200 AD). Major spheres of Celtic influence are circled.

The subsequent Unetice culture of the Upper Danube and Upper Rhine basins (**Figure 1**) shows a continuity of R1b1a1 haplotypes presumably contributed by the Beaker/Yamna admixture (Allentoft et al., 2015) and I2a subclades of Mesolithic hunter-gathers from the Balkans (Mathieson et al., 2018). This suggests that the Unetice culture served as a local nucleation center for agriculture, horse-assisted trade, prospecting, and metallurgy, probably speaking languages ancestral to Germanic and proto-Italo-Celtic (R1b-L11). The lack of DNA samples and complex genetic analyses from Tumulus and Urnfield groups prohibits us from observing a direct continuum from the Unetice people to the more recent haplogroups strongly associated with Celtic populations of Irish and Scottish (R1b-M222) origins, Germanic populations from the Rhine valley (R1b-S21/U106), Italic populations of the Liguria coast (R1b-S28/U152), or Gascons, Basques, and Catalans (R1b-DF27). The Hallstatt culture (800–450 BC), centered around the Alps (R1b-Z36), and its western successor, the La Tene culture (450–100 BC), are considered the first classical Celtic cultures that contributed to Gaul and Briton lineages in France (R1b-L21) and Celtiberian lineages in Spain (R1b-DF27), but substantial genetic gaps yet need to be filled before these conclusions can be ascertained.

### Early Archaeobotanical Evidence From Excavated Sites

Neolithic European settlements present in Europe since the migration of Anatolian farmers until early Bronze Age (*circa* 7000–1500 BC) hold the first archaeological records of humans gathering wild plants. The Late Mesolithic Lepenski Vir site within the Iron Gates gorge on the Danube (Serbia) produced early traces of wild grass grains such as einkorn and emmer wheat (y'Edynak and Fleisch, 1983), likely introduced by the Anatolian migrants *circa* 6000 BC (Mathieson et al., 2018). The lowest levels of the near-continuous Upper Paleolithic-Neolithic settlement at Franchti Cave (Greece) contained the largely uncharred carbonized seeds of field gromwell (*Lithospermum arvense* L.), alkanet (*Alkanna orientalis* L.), and bugloss (*Anchusa* sp.) (20000–11000 BC). Wild lentils (*Lens* sp.), vetch (*Vicia* sp.), pistachio (*Pistacia* sp.), almonds (*Prunus dulcis* Mill.), oats (*Avena* sp.), barley (*Hordeum spontaneum* K. Koch), and pear (*Pyrus amygdaliformis* Vill.) were recovered from the next Mesolithic layer (11000–7000 BC). The cultivated emmer wheat (*Triticum dicoccon* Schrank) also appeared at this site *circa* 6000 BC, similar to that of the Lepenski Vir settlement, followed by the cultivated two-row hulled barley (*Hordeum distichon* L.) and lentil (*Lens culinaris* Medik.) (Hansen and Renfrew, 1978). The comparative analysis of archaeobotanical data from 40 aceramic Neolithic sites from the eastern Mediterranean identified a near-identical founder crop package among these locations that consisted of emmer (*Triticum dicoccon* Schrank), einkorn (*Triticum monococcum* L.), hulled barley (*Hordeum vulgare* L.), flax (*Linum usitatissimum* L.), and four pulses—lentil (*Lens culinaris* Medik.), pea (*Pisum sativum* L.), bitter vetch (*Vicia ervilia* L.), and chick pea (*Cicer arietinum* L.). The adoption of this crop package led to an immediate reduction in vegetational taxon diversity at the settlements (Colledge et al., 2004).

It seems that an early tradition of collecting and using wild plants followed the same adoption pattern. Plants gathered at Franchti Cave and identified to the genus level (*Adonis, Alkanna, Anchusa, Calendula, Capparis, Colchicum, Fumaria, Lithospermum, Malva, Medicago, Phalaris, Salvia*, and *Silene*), could be considered medicinal by modern standards (Hansen and Renfrew, 1978). Moreover, transition to the early Neolithic settlements such as Theopetra (Greece), Revenia (Macedonia), or Sesklo (Greece) significantly increased variety of wild plant taxa at those sites, thus hinting at the diversity in early wild plant resources (Kotzamani and Livarda, 2018). When archaeobotanical data from 250 Neolithic sites was grouped into 22 European and east Mediterranean geographic regions (Coward et al., 2008), a clear east-west alignment in the direction of the Neolithic farming migration routes along the Danube and up the Rhine valley, but not the Mediterranean coast, was supported by multiple progressive plant gains (**Supplementary Table 1**). The diversity of wild plant findings showed a distinctive pattern of gains in Thalesian Greece, peaked in the Balkans (Bulgaria and Macedonia), and stabilized in the areas dominated by the Linear Pottery culture (LBK). Gathered plant gains included verbena (*Verbena officinalis* L.), agrimony (*Agrimonia eupatoria* L.*),* nightshade (*Atropa belladonna* L.), henbane (*Hyoscyamus niger* L.), ribwort plantain (*Plantago lanceolata* L.), poppy (*Papaver somniferum* L.*),* and wild strawberry (*Fragaria vesca* L.) Further migrations in the direction of Benelux, Scandinavia, and Southern Britain regions correlated with a divergent pattern of gathered species losses (Coward et al., 2008). The Mesolithic and Neolithic migrations into British Isles took place predominantly *via* Atlantic coastal routes (Farrell, 2015). As such, they were defined mostly by losses as compared to the traditional Neolithic Mediterranean plant assemblies (Coward et al., 2008). Notable gains here were puffballs (*Calvatia* sp.) from the Neolithic Skara Brae site (3180–2500 BC) in Orkney, Scotland (Watling and Seaward, 1976) and henbane (*Hyoscyamnus niger* L.) seeds in Grooved ware pots at the Balfrag settlement (2900 BC) in Fife, Scotland (Barclay et al., 1993). Meadowsweet (*Filipendula ulmaria* L.) was found at the Bronze Age cairn at Fan Foel, Carmarthenshire (Wales) as well as in a beaker at Ashgrove, Fife, and North Mains Strathallan (Scotland) (Henshall, 1963).

Although the putative health applications of these plants cannot be confirmed directly, their intended use can be implied by high frequency storage finds or detection in the gastrointestinal system of human remains. The examples included discovery of 8,000 perforated and intact seeds of purple gromwell (*Lithospermum purpurocaeruleum* L.) present within the same pot, or 22,400 seeds of common gromwell (*Lithospermum officinale* L.) in a goblet within a larger pot, dated to the Neolithic Cucuteni-Trypillia culture (Solcan et al., 2014). More than 54,000 seeds of pigweed (*Chenopodium album* L.) were found in a pot from the Neolithic settlement of Niederwil (Switzerland), as well as in the intestines of seven European Iron Age bog bodies (Behre, 2008). This is also true for hazelnut (*Corylus* sp.) and crab apple (*Malus* sp.) found in many in Neolithic assemblages and used to supplement everyday diets (Kirleis et al., 2012).

No significant changes in gathered wild plant profiles were observed in the findings from the 15 late Neolithic settlements of the Funnel Beaker culture from northern Germany (Kirleis et al., 2012) or six late Neolithic excavation sites attributed to Baden and Jevisovice groups in eastern Austria (Kohler-Schneider and Caneppele, 2009). However, the next two archeological horizons in Europe that were dominated by pastoral populations in the east (Corded Ware) and the west (late Bell Beaker), relied on different agricultural strategies and focused on single isolated farmsteads that often did not leave a distinct signal of cultivated and gathered wild plants. In those rare instances when these settlements could be identified as in case of the Engen-Welschingen “Guuhaslen” site, a similar set of wild gathered plants has been noted that also included ruderals nettle (*Urtica dioica* L.), hemlock (*Conium maculatum* L.), white violet (*Viola alba* Besser), elderberry (*Sambucus nigra* L. and *Sambucus ebulus* L.), and broadleaf plantain (*Plantago major* L.) (Lechterbeck et al., 2014). The history of plant assemblages from graves and megalithic tombs due to intentional activities toward their putative ritual and therapeutic use is lacking.

The east to west Paleogenetic trends of early Europe coupled with the emerging archaeobotanical pattern would suggest that the flora of the Thessalian Greece—Thracian Bulgaria and Macedonia—Central Europe axis along the Danube migratory route constituted the core pharmacy of proto-Indo-European medical traditions, predating the diversification of tribes into Italic, Celtic, Germanic, and Slavic. As history went on, this core tradition would be further diversified by the evolution of distinct ethnic groups, local oral knowledge, and plants gained and lost due to changes in environment. Ultimately these core plants, became fixed or standardized as “European medicine” by the written tradition of Greece and Rome as a result of Romanization, with small vestiges surviving in folklore and oral tradition.

### Proto-Celtic Cultures of Pre-Roman Conquest (800–275 BC)

The proto-Celtic tribes reached the area of the upper Danube and Rhine basins around 2500 BC. It is generally agreed that the first Celtic groups distinguished themselves from earlier Urnfield (proto-Celtic) and Villanova (proto-Etruscan) people *ca.* 800 BC through a succession of Hallstatt C-D and La Tene cultures centered around iron trade and salt mines of Hallstatt and Hallein (Dürrnberg) (**Figure 1**). This is the era of complex societies that ensured stability of bronze and later iron trade after the collapse of Southwest Asian and Mediterranean sources (Thurston, 2009). Despite numerous archeological records of graves, there are only a small number of Hallstatt and La Tene period settlement sites known, of which only a few have been excavated. Archaeobotanical remains from the Eberdingen-Hochdor site (600–400 BC) contained a few cultivated and wild plants, including carrot (*Daucus carota* L.), wild strawberry (*Fragaria vesca* L.), celery (*Apium graveolents* L.), camelina (*Camelina sativa* L.), parsley (*Petroselinum crispum* Mill.), hazel (*Corylus avellane* L.), and dyer's weed (*Reseda luteola* L.) (Stika, 1996). The first certain identification of *Cannabis* in Europe was also in the Hallstatt-period *Fürstengrab* of Hochdorf near Stuttgart, dated to *ca.* 500 BC (Merlin, 2003). While the use of opium, mandrake, and henbane were well documented in the Greco-Roman world prior to contact with the Celts, the ritual use of *Cannabis* was not a widespread practice within Greek and Roman societies, and could be likely attributed to direct early Indo-European influences of the Linear Pottery culture situated between Prut and Dnister rivers in Ukraine (Larina, 1999).

True botanical imports, including plants that were difficult to cultivate north of Alps such as black pepper (*Piper nigrum* L.), nutmeg (*Myristica fragrans* Houtt.), sesame (*Sesamum indicum* L.), or cumin (*Cuminum cyminum* L.) were found predominantly within Roman military camps in the Rhine frontier zone (Livarda, 2011). A similar observation was made from the archaeobotanical assemblages of Roman Britain, where imports (fig, mulberry, grape, olive), herbs (coriander, celery, dill, fennel, summer savory, and marjoram), and oils (black mustard, hemp) were much more common in major military sites and towns, especially in the southeast. Fruit (apple, pear, cherry, plum, damson, and walnut) and vegetable (carrot, cabbage, turnip, parsnip, and leaf beet) findings were better represented at Roman era rural sites, suggesting that cultivation was taken up by common people, and that some crops (*e.g.* apple) were present in their native wild form in the Late Iron Age. Dates, almonds, pine nuts, lentils, mulberry, and grapes were also found in the ceremonial context as votive offerings exclusively in Roman London temples, burials, and shrines (Veen et al., 2008). Since no imported Greek and Roman vessels and plants were found in Celtic rectilinear enclosures (known as *Viereckschanzen*) spread across Czech Republic to France and dated to 200–100 BC (Murray, 1995), this data indicated that Celtic culinary (and likely herbal) practices had little Roman influence until the end of the 1st century BC. Moreover, the first archaeobotanical records of imported non-native plants do not occur until after the Roman conquest of Britain (43 AD.) These included seeds of olive (*Olea europaea* L.), celery (*Apium graveolens* L.), coriander (*Coriandrum sativum* L.), and dill (*Anethum graveolents* L.) from the high status Silchester site, dated to 50 AD (Lodwick, 2014).

## Written Records From the Era of Roman Conflict (275 BC–476 AD)

Gallic expansion into Italy *ca.* 500 BC penetrated the Po valley and culminated in the battle of Allia and sack of Rome (390 BC). Boii and other Cisalpine Gaulish tribes often allied with Etruscan and Carthaginian armies against Rome, and their social organization and military tactics were extensively recorded by Roman writers. The military conflict intensified after the defeat of Carthage in 202 BC and resulted in annexation of *Gallia Cisalpina* in 192 BC, *Gallia Transalpina* in 121 BC, Gaul in 52 BC, and Britannia in 43 AD (**Figure 1**). It is speculated that capture of Rome and the Great Celtic expansion that followed were due to population pressure, political instability, and to establish secondary Gallic states of Tylis in Thrace (279–212 BC) and Galatia in central Anatolia (279–64 BC). Here, the knowledge of traditional Celtic medicine was recorded for the first time in several Greco-Roman herbal manuscripts focused on the medicinal flora of the Eastern Mediterranean region.

### Dioscorides—*De materia medica* (*ca.* 40–90 AD)

**Figure 2:**
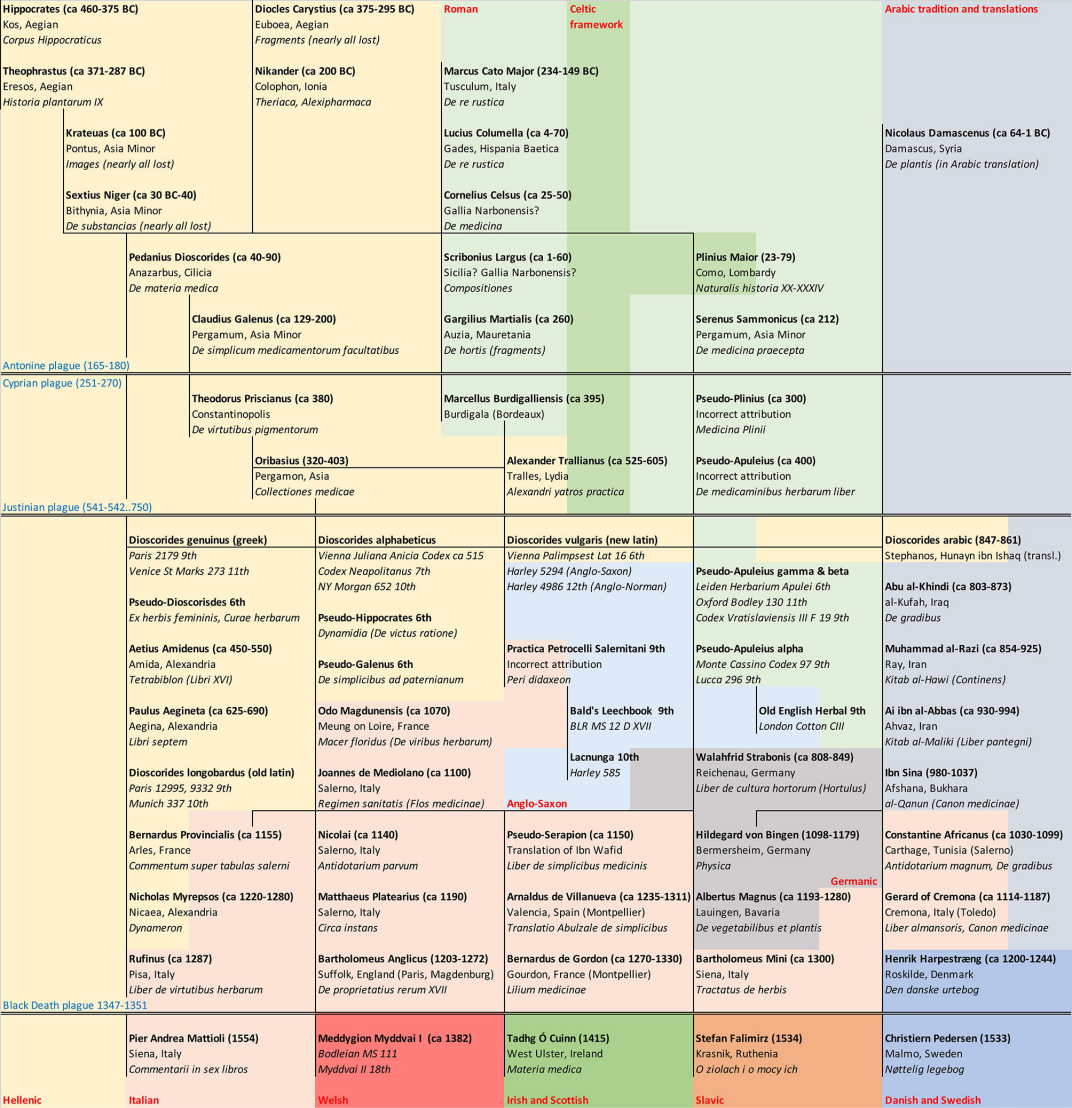
Chronological outline of the European herbal tradition. Notable gaps in knowledge exist due to historic epidemics that drastically reduced the population of Europe. The Celtic herbal framework was initially recorded the Roman authors, and was heavily influenced by the Salernian medical traditions, ultimately manifesting in Meddygon Myddfai I.

Pedanius Dioscorides, a Greek physician possibly employed in the Roman army and a native of the Roman province of Cilicia situated south of Galatia in Asia Minor, wrote a five-volume treatise on the preparations, strengths, and dosage of 600 herbs utilized in some 1,000 medicines. Adding an additional 100 plants over the *Historia Plantarum* of Theophrastus, he ignored classification using botanical characteristics in favor of direct medicinal properties and uses of herbs. *De materia medica* directly influenced the writings of the Greek physicians Galen (*ca.* 129–210 AD, *De simplicibus*), Theodorus Priscianus (*ca.* 380 AD, *De virtutibus pigmentorum*), Oribasius (320–403 AD, *Collectiones medicae*), and many more authors. Although the original Greek version of Dioscorides was continuously inscribed for the next several centuries, the text was also rearranged in the alphabetical order (i.e. *Vienna Juliana Anicia Codex ca.* 515 AD) and translated into Latin on multiple occasions. The early Latin translation was designated as *Dioscorides longobardus*, while the later translations were based upon the alphabetical version of the manuscript and often combined with information extracted from the Pseudo-Apuleius herbal (*Dioscorides vulgaris*) (Collins, 2000). Subsequent copies, interpretations, and translations of these manuscripts laid the foundation of the European herbal tradition of the 8^th^–14^th^ centuries (Singer, 1927) (**Figure 2**).

Dioscorides owes his universal acceptance to the development of an empirical tradition of herbal remedies throughout the Mediterranean region (Nissen, 1958). In the analysis of plant locations mentioned in his work, 67 refer to Asia Minor, 43 to the Arabic world, 38 to Africa, 30 to Greece and the Balkans, 12 to Italy, 8 to Spain, and 5 to the more northern territories (Denham and Whitelegg, 2014), suggesting that Dioscorides was only scarcely familiar with the plants of the Danube and Rhine basins. A section of Dioscorides, however, contains a collection of plant synonymous names (*Synonyma plantarum*) in multiple languages including Dacian and Gallic. Generally believed to be a later addition (Popa, 2010), the list nonetheless highlights the medicinal herbs of importance to the local regions of the empire as it generally excludes all exotic and cultivated plants, as well as animal and mineral treatments. The focus of the synonym list was likely to assist with identifying and sourcing local wild medicinal herbs in the regions outside of Greek and Latin tradition. A direct comparison between the plant lists that include geographically distinct Dacian and Gallic names may indicate a subset of native plants of high relevance to each area (**Table 1**). Several additional plants were also directly noted by Dioscorides as a part of the Iberian Celtic tradition, including eruca (*Erucastrum gallicum* Wild.) used as an aphrodisiac, and fennel gum (*Foeniculum vulgare* Mill.) as a more effective eye medicine than the plant juice. Finally, Celtic nard (*Valeriana celtica* L.) endemic to the Celtic Alps, was described as a tonic to the spleen, stomach, liver, and kidneys, that could be taken in wine with wormwood (*Artemisia absinthium* L.) as a “narcotic,” likely meaning a painkiller. Dioscorides reported very few superstitious practices, including the use of anchusa (*Echium* sp.) charms against bites.

**Table 1:** List of medicinal plants and their vernacular names from Celtic or Gallic herbal tradition, as compared to the Dacian plants of Dioscorides.

Botanical name	English name	Celtic (Gallic) name			Dacian name
		Dioscorides	Pliny	Marcellus	Dioscorides
*Achillea millefolium* L.	yarrow	beliucandas			diodela
*Acorus calamus* L.	sweet flag	piper apum			
*Adianthum* sp.	maidenhair fern				phithophthethela
*Ajuga iva* L.	bugle				dokela
*Alnus* sp.	alder			vernetus	
*Amaranthus blitum* L.	purple amaranth				bles
*Anagallis* sp.	pimpernel				kerker
*Anchusa italica* Retz.	anchusa				budalla
*Anethum graveolens* L.	dill				polpum
*Arctium* sp.	burdock				riborasta
*Aristolochia rotunda* L.	round birthwort	theximon			
*Artemisia absinthium* L.	wormwood				zeste
*Artemisia scoparia* Waldst	redstem wormwood				zired
*Artemisia vulgaris* L.	mugwort	ponem		biricumus	
*Arum maculatum* L.	arum				kourionnecum
*Asarum europaeum* L.	asarabacca	baccar			
*Aster amellus* L.	Italian aster				rathibida
*Atropa belladonna* L.	nightshade				koikolida
*Bryonia alba* L.	white bryony				dicotella
*Centaurium erythraea* Rafn	centaury				stirsozila
*Chelidonium majus* L.	greater celandine	thona			koustane
*Cochlearia anglica* L.	Engl. scurvygrass		britannica		
*Conium maculatum* L.	poison hemlock				zena
*Lagenaria siceraria* Molina	gourd				kinouboila
*Cynodon* sp.	dog's tooth grass				parithia
*Cynoglossum* sp.	hound's tongue				azila
*Daphne laureola* L.	spurge laurel	ousubim			
*Daphne mezereum* L.	daphne	ousubim			
*Dioscorea communis* L.	black bryony				priadela
*Dipsacus sylvestris* L.	wild teasel				skiare
*Dracunculus vulgaris* Schott	dragon lily			gigarus	
*Eryngium campestre* L.	eryngo				sikupnoex
*Erucastrum gallicum* Willd.	eruca	unknown			
*Fagus sylvatica* L.	beech		unknown		
*Ficaria verna* Huds.	lesser celandine				ebustrone
*Filix*	fern			ratis	
*Foeniculum vulgare* Mill.	fennel	sistrameor			
*Geranium sylvaticum* L.	cranesbill				aurumetti
*Gnaphalium* sp.	cudweed	gdasonen			
*Hedera helix* L.	climbing ivy	subites			arborria
*Helleborus niger* L.	black hellebore	laginum	unknown		prodiarna
*Hyoscyamus niger* L.	henbane	bilinuntiam			dielina
*Hypericum hircunum* L.	stinking tutsan				salia
*Iris foetidissima* L.	gladwin iris				aprus
*Isatis tinctorial* L.	dyer's woad		glastum		
*Juniperus communis* L.	juniper	jupicellusum			
*Limonium* sp.	sea lavender	iumbarum			
*Lithospermum tenuiflorum* Lf.	gromwell				gonoleta
*Lycopodium clavatum* L.	ground pine				kodela
*Huperzia selago* L.	clubmoss		selago		
*Matricaria recutita* L.	chamomile	lusta			amalusta
*Melissa officanlis* L.	lemon balm	merisimorion			
*Mentha pulegium* L.	pennyroyal	albolon			
*Mentha sylvestris* L.	horsemint				teudila
*Nepeta* sp.	catmint				tanidila
*Nymphaea alba* L.	water lily			baditis	
*Onobrychis caput-galii* Born	sainfoin				aniarsexe
*Panicum dactylon* L.	switchgrass				kotiata
*Papaver argemone* L.	prickly poppy	corna			
*Persicaria bistorta* L.	bistort				adila
*Plantago major* L.	plantain	tarbidolopion			skinpoax
*Polypodium vulgare* L.	polypody				karopithla
*Portulaca oleracea* L.	pulsane				lax
*Potamogeton compressus* L.	pondweed				koadama
*Potentilla reptans* L.	creeping cinquefoil	pempedula			probedula
*Quercus robur* L.	oak		unknown		
*Ranunculus* sp.	buttercup			blutthagio	
*Rosmarinus officinalis* L.	rosemary				dracontos
*Rubus canescens* DC.	wooly blackberry				manteia
*Salvia horminum* L.	annual clary				hormea
*Sambucus ebulus* L.	dwarf elder	ducone		odocos	olma
*Sambucus nigra* L.	elderberry	scobie			seba
*Samolus valerandi* L.	water pimpernel		samolus		
*Solanum nigrum* L.	black nightshade	scubulum			
*Thymus* sp.	thyme			gilarus	mizeia
*Trifolium* sp.	clover			visumarus	
*Tussilago farfara* L.	coltsfoot			calliomarcus	asa
*Urtica dioica* L.	nettle				dyn
*Valeriana celtica* L.	celtic nard	unknown			
*Verbascum* sp.	mullein				diesapter
*Veronica officinalis* L.	speedwell	sapana			
*Viscum album* L.	mistletoe		unknown		
*Withania somnifera* L.	groundcherry				kykolis

### Pliny’s Account of Gallic Druids (23–79 AD)

Pliny the Elder, a Roman author born in Como, Lombardy, described the Celtic healers, or druids as “magicians” and “priests” and talked of their fondness for plants (*Naturalis Historia,* Book XIV). Special attention was given to mistletoe (*Viscum album* L.), especially when grown upon the English oak (*Quercus robur* L.), as medicine and religious sacrament of the Celts. Pliny described other Celtic plants including *glastum* or dyer's woad (*Isatis tinctoria* L.) as a source of blue dye, the use of beech (*Fagus sylvatica* L.) ashes for reddish hair, hellebore (*Helleborus niger* L.) as arrow poison, and *selago* similar to savin (*Huperzia selago* L.) for eye infections. Even though much of Pliny's material came from Theophrastus or from shared sources, he described several new herbs including *britannica*, a plant that grew on the islands off the Frisian coast and was used as a cure for scurvy, quinsy, and snake bites. This name could be possibly attributed to the extremophile English scurvygrass (*Cochlearia anglica* L.) rich in vitamin C (Nawaz et al., 2017) (**Table 1**).

The herbal practices of Hellenized Anatolia prioritized a rational approach to making and prescribing remedies, therefore deliberately avoiding magical formulas. Latin herbal writers, however, continuously mention magical cures in the form of spells, charms, and incantations, even while being hostile to these practices, as in the case of Pliny. Among 27 magical remedies listed in his work, only four could be attributed to Greek and Latin origin. One of them, attributed to use of reseda (*Reseda alba* L.) to treat inflammation, originated in the vicinity a Roman colony Ariminum (Gaillard-Seux, 2014), in the area of Northern Italy held by Celtic tribes since the 6^th^ century BC. The majority of other formulas, as attested by Pliny, were supplied by unknown *magi*. On the other hand, *Codex Ardmachanus*, a 9^th^ century Irish manuscript written mainly in Latin, specifically applied this term to those who in the Irish tradition were called *Druadh* (Skene, 1886). It seems that Pliny's sources have been referring to Celtic *magi* (druids), and their magical formulas were incorporated into the Latin works by direct geographical proximity and societal overlap of Roman and Celtic cultures after 250 BC.

### Marcus Cato—*De re rustica* (*ca.* 234–149 BC)

A separate branch of herbal knowledge rather independent from Theophrastus, Pliny, and Dioscorides works was initiated by Marcus Cato of Tusculum, known for his conservatism and opposition to Hellenization. He recorded the folk knowledge related to the agricultural and herbal tradition of the Italic and Celtic tribes, including the use of offerings, charms, and incantations for healing practices. Cato advised on the use of cabbage (*Brassica oleracea*) to heal multiple inflammatory and gastrointestinal disorders, the use of urine collected after cabbage ingestion, and underlined the higher therapeutic potency of wild cabbage compared to its cultivated relatives.

### Celsus—*De medicina* (*ca.* 25 BC–50 AD)

Cornelius Celsus was most likely associated with *Gallia Narbonensis*, which became a Roman province in 121 BC. This relationship was suggested based on his remarks about a very specific vine (*marcum*) which, according to Pliny, was native to Narbonese Gaul (Langslow, 2000), as well as close familiarity with Gallic hunting poisons and their practices to cauterize the blood vessels. The writings of Celsus were contemporary to Pliny and Dioscorides, and took place shortly before Claudius, the “Gallic” emperor, addressed the senate to allow Gallic aristocrats to enter the Roman senatorial class in 48 AD. This idea was abandoned after the Gallic revolts of 68–70 AD, and even Tacitus, a Gaul himself, believed that the oppression of northern Gauls was a necessary evil (Woolf, 2000). At the same time, the last major stronghold of Celtic druids at Anglesey, Wales was destroyed and brought to the Roman empire in 60–78 AD. Although Celsus provided an extensive description of using wild and pot-cultivated herbs for medicinal purposes, none of them is explicitly stated by him to be attributed to a Celtic tradition.

### Scribonius Largus—*Compositiones* (*ca.* 1–60)

Scribonius Largus was believed to accompany Claudius on the British campaign of 43 AD and assembled his own version of herbal prescriptions similar to Cato and Celsus. His birthplace remained uncertain with contradicting sources pointing to Sicily or *Gallia Narbonensis*, and he clearly shared knowledge or some common sources with Celsus as evident from their descriptions of Theriaca and Mithridatic remedies (Baldwin, 1992). Following his predecessor's trends, Scribonius also preserved the superstitious and highly magical nature of many prescriptions, however unlike Celsus, he placed a strong emphasis on exact dosing of individual components, in addition to the general description of the herbal types and mixtures. Scribonius describes a variety of eye diseases which were among the most common afflictions in Gaul, and the use of the dissected ointment sticks (*collyria*) in preparation of an eye salve. This form of treatment (dry packaging in large batches) was rather unique to Gaul and the British Isles and could be possibly explained by limited and often difficult access to Eastern Mediterranean herbs required for these preparations (Baker, 2011).

### Marcellus Empiricus—*De medicamentis liber* (*ca.* 395–410)

The Celtic magical formulas found in the writings of Pliny, Cato, Celsus, and Scribonius peaked in the *De medicamentis liber* of Marcellus Empiricus. For example, Marcellus advises certain plants to be collected with the waning of the moon, the use of iron forbidden in digging or cutting the plant, requiring certain plants to be collected with the left hand. This is highly similar to Pliny's description of *Samolus valerandi* or water pimpernel. The marsh dwelling plant was said to be gathered by a fasted druid with his left hand (Stannard, 1973).

In addition to relying on previous writers, Marcellus clearly stated that the bulk of his recipes came from the local population (*sed etiam ab agrestions et plebeis*). He listed 12 Celtic plant names, ten of which were accompanied by a Greek or Latin synonym. These plants included *baditis* (water lily, *Nymphaea alba* L.), *biricumus* (mugwort, *Artemisia vulgaris* L.), *calliomarcus* (colt's foot, *Tussilago farfara* L.)*, gigarus* (snake lily, *Dracunculus vulgaris* Schott)*, gilarus* (thyme, *Thymus serpyllum* L.), *odocos* (elder, *Sambucus ebulus* L.), *ratis* (ferns, *Pteridophyta),* and *visumarus* (clover, *Trifolium* sp.). The two plants without synonyms were tentatively identified as *vernetus* or *viridis* (alder, *Alnus* sp.) and *blutthagio* (buttercup, *Ranunculus* sp.) (Stannard, 1973) (**Table 1**). Turning to polypharmacy, most of his recipes contained 10–20 plant constituents, and magical formulas, charms, and incantations formed an intrinsic part of his therapeutic strategies.

Marcellus’ work marked the turning point in our direct knowledge of Celtic herbal tradition, as the druids left no original or surviving writings, and the contemporary Roman culture experienced a drastic social and political decline following the Antonine (165–180 AD), Cyprian (251–270 AD), and Justinian (541–542 AD) plagues that killed as much as 30–40% of the population in the affected areas, and devastated the Roman army. From here, we can only discuss the surviving Celtic herbal framework within the fringes of the British Isles (Wales, Scotland, Ireland, and the Isle of Man).

## *Meddygon Myddfai* in the Red Book of Hergest (Shortly After 1382)

The demographic pressure from Germanic tribes along the Rhine and Danube frontiers, combined with Anglo-Saxon and Norman expansion into Brittany confined Celtic tribes to the western regions of the British Isles and Ireland. The advance of Christianity and associated Crusades consolidated Western Europe as a unified Christian force, and it seems its medical tradition followed suit. The herbal texts of Medieval Europe were dogmatically transcribed and translated from existing works, mostly different versions of Dioscorides and simplified Pseudo-Apuleius. This also included a series of translations from Arabic sources (al-Razi, Ibn-Sina, Haly Abbas) by Constantine Africanus and other writers. Medical schools of Salerno and Padua also maintained the Greco-Roman herbal tradition of the existing herbal manuscripts and their interpretations such as Odo Magdunensis (*Macer Floridus*), Matthaeus Platearius (*Circa instans*), John of Milano (*Regimen sanitatis*), Nicolai Salernitanus (*Antidotarium parvum*), and Rufinus (*Liber de virtutibus herbarum*) (**Figure 2**).

Away from the Mediterranean, the classical herbals and their translations were of ever decreasing relevance, which prompted a revision and fixation of a distinct and presumably independent vernacular herbal knowledge. It was the turn of the outskirts of Medieval Europe to develop their own herbals by synthesizing the classic sources and local tradition, as evident from reappearance of magic formulas, incantations, and vernacular plant names in these works. Three manuscripts that fall under this category include the 9^th^ century Anglo-Saxon Bald's Leechbook (*Medicinale anglicum*), the 12^th^ century Germanic *Physica* (*Liber simplicis medicinae*) by Hildegard of Bingen (1098–1179), and the 14^th^ century Welsh *Meddygon Myddfai* as a part of the Red Book of Hergest (Bodleian MS 111, shortly after 1382). The latter was said to incorporate the healing tradition of Rhiwallon Feddyg, the physician to Rhys Gryg from the Cymry Celtic kingdom of Deheubarth (*ca.* 1234). The manuscript is divided in two parts: *Myddfai I* (recipes 1–188 considered original to 1382) and *Myddfai II* (recipes 189–815 allegedly drawn by Iolo Morganwg from the continental herbal tradition (Luft, 2018). Both *Bald's Leechbook* and *Meddygon Myddfai* seem to contain larger amounts of magical formulas and superstitious treatments than the classical herbal manuscripts of Pliny, Dioscorides, and Galen, and more resemble the approach of Celsus, Scribonius Largus, and Marcellus Empiricus in incorporating magical elements and elaborate herbal preparations with multiple constituents. It is rather likely that these manuscripts shared common sources that to a large extent relied upon Marcellus Empiricus and Alexander of Tralles (*Alexandri yatros practica*) and appealed to personal experiences in discovering and testing the herbal remedies.

### Organization of *Meddygon Myddfai*

In contrast to Dioscorides, whose treatments were organized by substances (earlier Greek and old Latin manuscripts), or revised into an alphabetical order (later Greek and Latin translations), *Myddfai I* did not follow an obvious organizational structure and seemed to represent a loosely collected list of recipes that was developed over time. This also differed from the *Bald's Leechbook*, which preserved the classical head-to-toe order. The botanical descriptions in *Myddfai I* were often significantly reduced or eliminated altogether, suggesting that the manuscript was developed as a quick reference guide of directions (steps) to be performed by a person versed in the herbal tradition and medicine, rather than a medicinal text to be read and interpreted by a medieval scholar. Straightforward passages such as “*for an illness…, take a number of plants…, crush them in a vehicle medium., set aside…, have a patient use…*” differed drastically with other herbal collections, and did not intend to educate readers in the art of identification and collection of medicinal plants. Another two features that set *Myddfai I* aside from the contemporary and classical herbals were i) the use of numerous and diverse media for the preparation of herbal remedies, and ii) the emphasis on magic formulas, charms, and incantations. Both groupings garnered very little attention in past scholarly works, however they are more likely to preserve the original references and vernacular knowledge of Welsh belief systems and practices as it applies to Celtic medicine as a whole.

**Figure 3:**
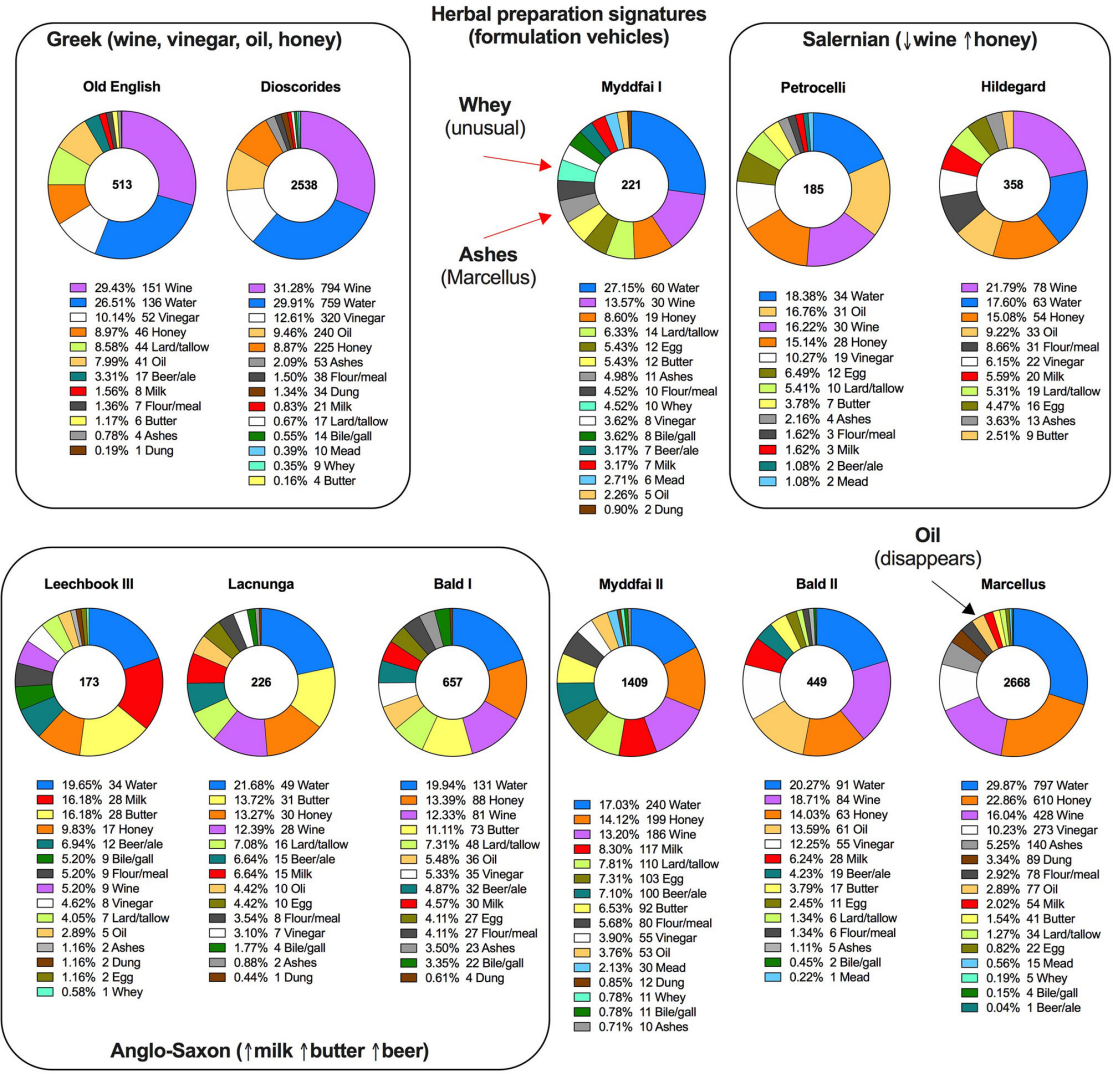
Herbal preparation signatures recorded in major texts of the European herbal tradition. The recipes in Myddfai I are unique in high frequency usage of whey (primarily goat milk whey) as a formulation vehicle for remedies.

Herbal preparation signatures can be easily evaluated in the herbal manuscripts by calculating the frequencies of certain vehicles (media) used to crush, dissolve, or extract plant botanical material during formulation of the remedies. As evident from Dioscorides, classical Greco-Roman herbal tradition relied heavily on using wine, vinegar, and oil in addition to water as media for preparation of plant remedies. This signature clearly survived in Pseudo-Apuleius (4^th^ century) and it's Old English translation (9^th^ century) despite multiple scribes, edits, rearrangements, and translation of the recipes. The signature is still evident in the work of Marcellus Empiricus (however, his formulations show limited use of oil and increased use of ashes), and multiple manuscripts of Italian and German tradition such as works attributed to Petrocelli (9^th^ century) or Hildegard (12^th^ century). It is interesting that *Bald's Leechbook II* manuscript also falls under this category, while *Leechbooks I, III*, and the *Lacnunga* maintained sets of remedies that used a different, presumably Anglo-Saxon, herbal preparation signature based on an increased use of milk, butter, and beer (**Figure 3**). The *Myddfai I* signature generally follows a Mediterranean tradition with several exceptions such as a decreased use of wine, and an increased use of whey (not frequent in other manuscripts) and ashes (similar to Marcellus Empiricus). *Myddfai II*, in concordance with stipulation of being a recent 18^th^ century compilation from multiple herbal sources, shows a mixed herbal preparation signature with multiple formulation media present at nearly equal frequencies, including an increased use of milk and beer typical to the Anglo-Saxon tradition and largely limited in *Myddfai I*. It would be very interesting to extend the analysis of the herbal preparation signatures to other herbal manuscripts from the European (Salerno, Montpellier, Padua) and Arabic tradition, as well as different versions of the same manuscript scribed in the geographically distinct areas and time periods.

### Plants of *Meddygon Myddfai I*

In an attempt to further distinguish the Welsh herbal tradition, we performed a systematic analysis of plant species and the target health conditions described in the *Myddfai I* manuscript (Oxford Jesus College MS 111) against herbal texts of various medical traditions (**Figure 2**). The correct identification of modern plant names in the text was challenging due to the cryptic nature of Middle Welsh, as well as possible mistakes during scribing and incorrect previous translations as noted in **Table 2**. Longer recipes such as 11, 12, and 19 contained the most errors in translation and corrupt phytonyms. For instance, common burnet (*Sanguisorba officinalis* L.) is not actually present in the Welsh text but is found in the English translations of recipe 19. Uncertain plant names of Welsh origin were further checked against Davies' *Welsh Botanology* (1813), Richard's *Antiquae linguae Britannicae thesaurus* (1815), and Pughe's *Dictionary of the Welsh Language* (1832). In some cases, the common name was too general to resolve, such a *redyn* for “fern” or *redega
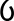
c* for “liverwort”. Plant names not found to match Dioscorides were also corroborated against other sources, including Dunbar's *A New Greek and English Lexicon* (1844) and the Natural History of Pliny.

**Table 2:** The list of *Myddfai I* plants and their health indications (recipes 1–188).

Latin name	English name	Middle Welsh name (*MM*)	Recipe(s)	Indication
*Achillea millefolium* L.	milfoil	uilff 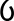 th	12, 16, 19, 121, 127, 132, 133, 136	fever, kidney stones, vomiting blood, worms, epistaxis, vomiting
*Agrimonia eupatoria* L.	agrimony	trydon, try 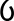	11, 12, 19, 20	pneumonia, fever, kidney stones, fertility
*Agrostemma githago* L.	corn cockle	tenteulys^1^ uendigeit	11	pneumonia
*Allium ampeloprasum* L.	leek	gennyn, kenin	100, 135	dog bite, vomiting of blood, fertility, snake bite, ulcers, whooping cough, pneumonia, deafness, headache, bone healing, boils, increase strength, flatulency, worms
*Allium sativum* L, (var. *ophioscorodon* (Link) Döll)	garlic (and viper's garlic)	craf, garllec	24, 56, 96, 97, 138	wounds, prevent fatigue, swelling, pain, epistaxis, proud flesh
*Allium ursinum* L.	ramsons	craf y geiuyr	53	abdominal complaints
*Anagallis arvensis* L.	pimpernel	diwythyl	15, 17, 20, 21, 53	fever, abdominal complaints, fertility, menorrhagia
*Apium graveolens* L.	smallage (celery)	ysmalaes, api 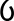 m	116, 124, 128	smallpox, calming, ague
*Aquilegia vulgaris* L.	columbine	columbina	19,	kidney stones
*Arctium minus* Hill	small burdock	kynga 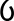 man	12, 21	fever, menorrhagia
*Aristolochia rotunda* L.	round birthwort	hennllydan	11, 12, 42, 43, 45, 51	pneumonia, fever, toothache, inflammation, snake bite
*Artemisia abrotanum* L.	southernwood	bryt 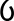 n	102, 124	insanity, palsy
*Artemisia absinthium* L.	wormwood	wermot	12, 13, 65, 120, 128	fever, destroy fleas, general wellness, snake bite, ague
*Artemisia dracunulus* L.	taragon	dragrans	126	worms
*Artemisia vulgaris* L.	mugwort	gannwreid	12, 15, 25, 38, 51, 56, 66, 110, 111, 112, 128, 144	fever, worms, carbuncle, prevent fatigue, destroy flies, snake bite, difficult childbirth (ritual), ague
*Asarum europaeum* L.	asarabacca	alannon	11	pneumonia
*Asplenium scolopendrium* L.	hart's tongue	dauot yr hydd	60, 78	anaphrodisiac, general wellness
*Avena sativa* L.	oats	keirch	13, 53	poultice, dietary
*Bellis perennis* L.	daisy	hygat y dyd	11, 12, 64, 102, 161	pneumonia, fever, worts, insanity, tumor
*Betula pendula* Roth	birch	uedl 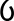 yn	19, 50	kidney stones, impotency,
*Borago officinalis* L.	borage	glessyn	15, 17, 19	fever, abdominal complaints, kidney stones
*Brassica oleracea* L.	cabbage (red and green)	ca 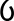 l (coch)	12, 99	fever, nettle rash
*Brassica rapa* L.	turnips	eruin	108, 117	worms, surfeit?
*Calluna vulgaris* L.	heath	gruc	11, 12, 116	pneumonia, smallpox
*Cannabis sativa* L.	hemp	kywarch	12	fever
*Capsella bursa-pastoris* L.	shepherd's purse	ph 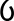 rs y bugeil	166	heartache
*Carum carvi* L.	caraway	gara 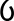 yt, kara 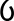 n, Iarderv	11, 166	pneumonia, heartache
*Centaurea nigra* L.	knapweed	benngalet	12, 38, 45	fever, carbuncle, snake bite
*Centaurium erythraea* L.	centaury	ysca 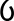 l crist, centa 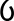 rya^1^	12, 114, 115	fever, kidney pains, extreme thirst
*Chelidonium majus* L.	celandine	llyssey y wennol	170	eye problems
*Chenopodium album* L.	goosefoot	roec, dudren	21, 166	menorrhagia, heartache
*Cirsium vulgare* Savi	common thistle	ysgall	122	constipation
*Conium maculata* L.	hemlock	kygget	11	pneumonia
*Conopodium majus* Gouan	earthnut	bywi	12, 15, 17	fever, abdominal complaints
*Coriandrum sativum* L.	coriander	koliandr 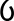 m	128	ague
*Corylus avellana* L.	hazel	coll	186	cleaning teeth
*Crataegus monogyna* Jacq	white thorn	yspydat	36	worms
*Crocus sativus* L.	saffron	saffyr	57, 58, 146	remove drunkenness, induce happiness
*Datura stramonium* L.	thorn apple (dorycnion)	vennwen	42	toothache
*Digitalis purpurea* L.	foxglove	ffiol y ffrud	53, 74	abdominal complaints, tumors
*Empetrum nigrum* L.	crake berry	grygyon	11, 12	pneumonia, fever
*Eryngium maritimum* L.	eringo	mor gelyn	52	toothache
*Eupatorium cannabinum* L.	hemp agrimony	vedun chwer 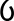	56, 78, 107	prevent drunkenness, general wellness, cough
*Ficus carica* L.	figs	ffigys	137	poison
*Filipendula ulmaria* L.	meadow sweet	erch 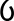 reid, uedlys^1^	40	pneumonia, fever, kidney stones, hemorrhage
*Filix*	fern	redyn	98, 155	burns, hemorrhoids
*Foeniculum vulgare* Mill	fennel	ffenigyl	15, 79, 96, 100, 120, 123, 170	fever, general wellness, swelling, pain, dog bite, snake bite, digestion, eye problems
*Fragaria vesca* L.	strawberry	syui	10	eye problems
*Fraxinus excelsior* L.	ash	onn	31	deafness
*Fumaria officinalis* L.	fumitory	m 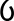 c y dayar	13	fever
*Galium odoratum* L.	woodruff	udrot	11, 12, 79	pneumonia
*Galium verum* L.	yellow bed straw	kylyon, keulon	35, 162	spider bite, swelling
*Geranium robertanium* L.	herb Robert	troet rud	11	pneumonia
*Geum urbanum* L.	avens	uab coll	11, 12, 21, 41	pneumonia, menorrhagia, hoarseness
*Glechoma hederacea* L.	ground ivy	ganwreid rydega 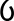 c, eido y dayar, eidral^1^	15, 19, 72, 100, 129, 145	fever, kidney stones, eye problems, dog bite, ague
*Hedera helix* L.	ivy	eidor 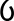 c	29, 30, 98	skin problems, toothache, burns
*Helleborus foetidus* L.	stinking hellebore	hylithyr	17	abdominal complaints
*Hepatica*	liverwort	redega 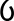 c	17, 19	abdominal complaints, kidney stones
*Hordeum vulgare* L.	barley	heid	33, 111, 163, 168	plaster, dietary, worms, boils,
*Huperzia selago* L.	fir clubmoss	thar 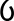 y mynyd	20	fertility
*Hypericum androsaemum* L.	tutsan	greulys (uendigeit) ^1^	12 (English only), 15, 19, 95, 101	fever, kidney stones, inflammation
*Hypericum perforatum* L.	St. John's wort	erinllis	12, 19, 20, 38, 41	fever, kidney stones, fertility, carbuncle, hoarseness
*Juglans regia* L.	walnut	coll ffrenghic	36	worms
*Knautia arvensis* L.	field scabious	bennlas	12, 45	fever, snake bite
*Lactuca* sp.	lettuce	g 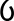 ylaeth, letus	80, 157	general wellness, fertility
*Lamium purpureum* L.	red nettle	dyna coch	12, 38, 51, 164	fever, carbuncle, strangury
*Laurus nobilis* L.	bay	dodeit	17, 19	abdominal complaints, kidney stones
*Legousia speculum-veneris* Fisch	corn bell flower	drycheigyauc	22	quinsy
*Lemna* sp.	duckweed	linat	94, 103	abdominal complaints, constipation
*Leonurus cardiaca* L.	motherwort	vamyls	19	kidney stones
*Lepidium latifolium* L.	pepperwort	bybyrllys^1^	12	fever
*Ligustrum vulgare* L.	privet	rysswyd	12	fever
*Lilium candidum* L.	white lily	lili 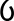 m g 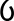 ynn	98, 118	burns
*Linum usitatissimum* L.	linseed, flax	llin	7, 19	head wound, wounds
*Lithospermum* sp.	gromwel	gr 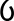 myn	19, 151	kidney stones
*Lonicera caprifolium* L.	honeysuckle	g 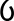 ydwyd	30	toothache
*Malus domestica* Borkh.	apple	aualeu, aual	14, 15, 55	aprient, ritual
*Malva sylvestris* L.	mallows	hokys	15, 17, 59, 143	fever, hemorrhoids, abdominal complaints, general wellness
*Matricaria* sp.	chamomile	amrannwen	39, 125	wounds, reptiles in stomach
*Melissa officinalis* L.	lemon balm	g 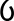 enyn	116	smallpox
*Melittis melissophylum* L.	bastard balm	wenenllys uan	12, 21	fever, menorrhagia
*Muscus*	moss	misyc, unsyc	15, 17	fever, abdominal complaints
*Myrica gale* L.	sweet gale	vrine	17	abdominal complaints
*Origanum dictamnus* L.	dittany	ditaen	126, 131, 160	worms, poison, pain
*Oxalis acetosella* L.	wood sorrel	suryon y coet	15, 34	fever, headache, joint pain,
*Papaver somniferum* L.	poppy	pabi	49	sleep
*Persicaria amphibia* L.	amphibious persicaria	granwreid benngoch	12, 15, 19, 20	fever, kidney stones, fertility
*Petroselinum crispum* Mill.	parsley	persli	164	strangury
*Pimpinella anisum* L.	anise	ennyd	11	pneumonia
*Piper nigrum* L.	pepper (white and black)	pybyr	82, 100, 128, 138, 151, 157	general wellness, ague, proud flesh, kidney stones, fertility, bone healing
*Plantago major* L.	plantago major	erllyrat, plantaen	110, 127, 128, 161, 162, 163, 166	snake bite, worms, ague, tumor, swelling, boils, heartache
*Potentilla reptans* L.	creeping cinquefoil	ganwreid uelen	20	fertility
*Primula vulgaris* Huds.	primrose	briallu	68	loss of reason or speech
*Prunella vulgaris* L.	self-heal	wennela 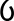 c, ueidya 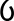 c	38, 42	carbuncle, toothache
*Prunus persica* L.	peach	persig	108	worms
*Prunus spinosa* L.	blackthorn	eiryn	167	dyspepsia
*Quercus* sp.	oak	derwhyden, keginderw	29, 166	skin problems, heartache
*Ranunculus ficaria* L.	lesser celandine	celidonia	96, 170	swelling, pain, eye problems
*Raphanus sativus* L.	radish	raphan, (r)hadigyl	120, 156	snake bite, dog bite
*Rubia peregrina* L.	little field madder	wreidr 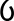 d l 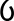 yt	19	kidney stones
*Rubia tinctorum* L.	madder	wreidr 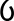 d	11,12	pneumonia, fever
*Rumex conglomeratus* Murray	sharp dock	turth, twrch	11	pneumonia
*Rumex* spp.	docken	tuaol	26, 42	abscess, toothache
*Ruscus aculeatus* L.	butcher's broom	iewydd, ieuta 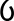 t^1^	12, 15	fever
*Ruta graveolens* L.	rue	rut	110, 111, 113, 120, 128, 137, 160	snake bite, worms, swelling, pain, ague, poisoning, anaphrodisiac
*Salix* sp.	willow	helic	165	warts
*Salvia officinalis* L.	sage	saluia	102, 138	insanity, proud flesh
*Salvia sclarea* L.	clary clary	llygeit crist	59	anaphrodisiac
*Sambucus ebulus* L.	dwarf elder	gruelys war/ua 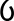 r	12, 15	fever
*Sambucus nigra* L.	elder	ysga 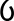	15, 16, 36, 67	fever, hemorrhoids, worms, snake bite
*Samolus valerandi* L.	water pimpernel	glaerllys	19	kidney stones
*Saxifraga granulata* L.	saxifrage	saxifraga (tormaen)	151	kidney stones
*Scandix pecten-veneris* L.	shepherd's needle	greithic	11, 24	pneumonia, wounds
*Scapania undulata* L.	river startip	gynglenyd	17, 106	abdominal complaints, liver disease
*Secale cereale* L.	rye	ryc	107, 138, 162	cough
*Sedum telephium* L.	orpine	ganwein	12, 20, 21	fever, fertility, menorrhagia
*Sinapis alba* L.	mustard	m 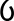 start	139	expel cold humors, snake bites, toothache, menorrhagia, digestion, colic, hair loss, tympanitis, dimness of sight, skin problems, palsy
*Solanum dulcamara* L.	bittersweet nightshade	elinya 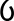 c	36	worms
*Solanum nigrum* L.	black nightshade	morella	163	boils
*Stachys officianlis* L.	betony	danna 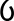 c, danhogen	5, 12, 19, 34, 42, 105, 121, 130	head wound, fever, kidney stones, headache, joint pain, toothache, epistaxis, vomiting blood, vomiting
*Symphytum officinale* L.	comfrey	chwefyrdan, consolida maior	26, 169	abscess, bone healing
*Taraxacum officinale* L.	dandelion	deint ylle 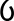	13, 19, 23, 34	fever, kidney stones, exfoliation of the skull, headache, joint pain
*Taxus baccata* L.	yew	ia 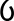 n	20	fertility
*Tragopogon dubius* Scop.	yellow goat's beard	g 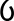 reid yr erweint	11, 12, 20	pneumonia
*Triticum* spp.	wheat	g 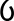 enith	13, 34, 61	plaster, dietary
*Urtica dioica* L.	nettles	dynhaden	132	epistaxis
*Valeriana officinalis* L.	valerian	llysseu cad 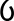 ga 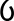 n	26	abscess
*Veronica officinalis* L.	speedwell	ieuda 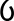 t	17, 38	abdominal complaints, carbuncle
*Viola odorata* L.	violet	violet, uiolet	5, 7, 55, 149	head wound, ritual

1 Translation of plant names in recipes 11, 12, 15, 19 is corrupted.

- *Tenteulys uendigeit* (*Agrostemma githago* L.) could be a corruption of *greulys fendigaid* for tutsan (Hypericum androsaemum L.).

- Ysca
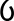
l crist (Centaurium erythraea L.) could also indicate St. John's wort (Hypericum perforatum L.).

- *Erchwreid/orchwreid* (*Filipendula ulmaria* L.) (11,12,19) could be a corruption of *olchwraidd* (Sanicula europea L.) *Uedlys* (40) is correct.

- *Ganwreid rydega
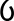
c* (*Glechoma hederacea* L.) (15) is actually creeping persicaria (*Persicaria* sp.); *eidral* (100) could indicate *Hedera helix; eido y dayar* is correct.

- Twrch (Hypericum androsaemum L) (12) is likely sharp dock (Rumex conglomeratus Murray).

- *Bybyrllys* (*Lepidium latifolium* L.) is an incorrect translation (12) to peppermint.

- Ieuda
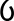
t (*Ruscus aculeatus* L.) is actually speedwell (*Veronica officinalis* L.).

**Figure 4:**
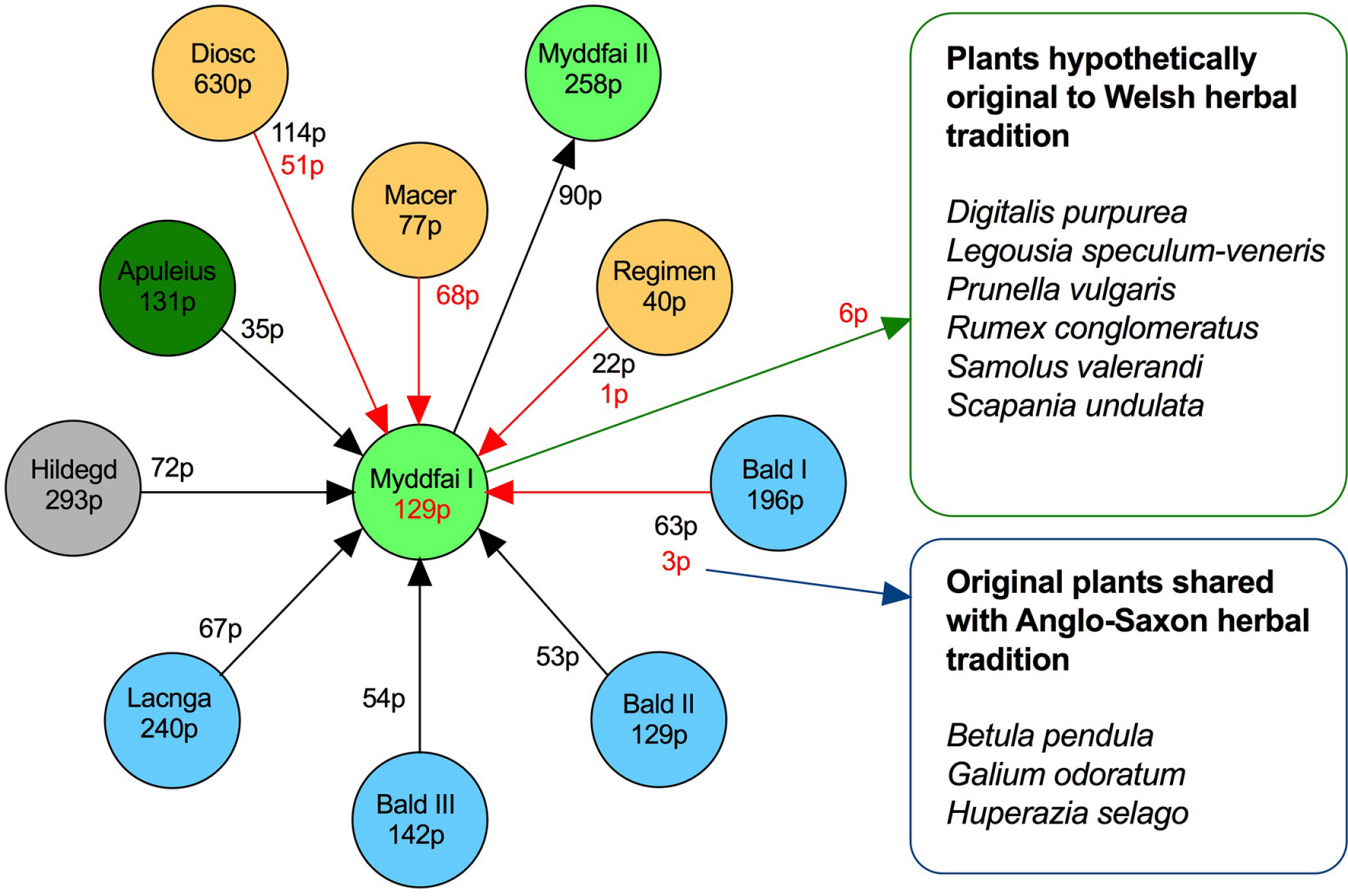
Plant (p) species overlap between Myddfai I and other traditional herbal sources. Arrows indicate hypothetical influence (red) or overlap (black).

Direct counts of plant species listed in these herbal manuscripts indicated that Macer Floridus *De viribus herbarum* shared the most extensive overlap with *Myddfai I* (out of 77 plant species found in Macer Floridus, 68 (88%) were also mentioned in *Myddfai I*) (**Figure 4**). Approximately 114 plant species overlapped between Dioscorides' *De Materia medica* and *Myddfai I* as well, and the former contributed a set of 51 plant species not found in Macer Floridus to *Myddfai* I recipes. One more plant species (primrose, *Primula vulgaris* Huds.) was mentioned in *Regimen sanitatis*, thus bringing the total number of *Myddfai I* plant species of classic Mediterranean herbal tradition to 120. Of those, only 5–6 species had to be imported in dried form from the warmer regions, suggesting that the manuscript was intentionally developed to exclude the most exotic species due to cost and/or lack of availability. The remaining nine plant species could be hypothetically allocated to the herbal medical tradition of the British Isles and Ireland. Three plant species were derived from the *Bald's Leechbook* or the common shared sources between the two manuscripts, including silver birch (*Betula pendula* Roth), woodruff (*Galium odoratum* L.), and fir clubmoss (*Huperzia selago* L.). The remaining six plants (**Table 3**) (and their various names), which were not mentioned in any text but *Myddfai I,* are discussed briefly below.

**Table 3:** *Myddfai I* plants hypothetically original to the herbal tradition of British Isles and Ireland.

Tradition	Welsh	Welsh	Welsh	Welsh	Welsh	Welsh	Anglo-Saxon	Anglo-Saxon	Anglo-Saxon
**Botanical print**	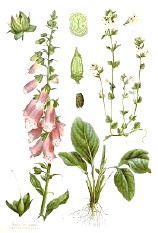	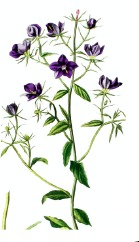	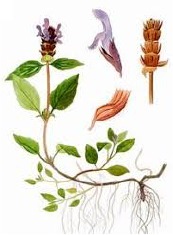	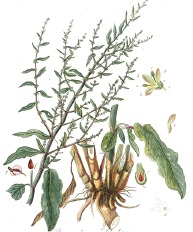	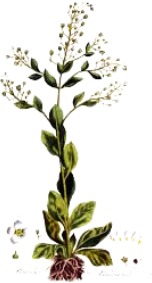	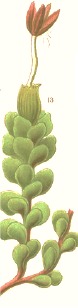	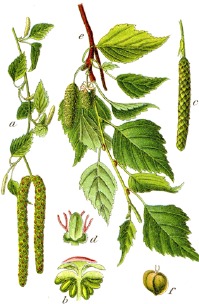	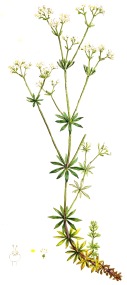	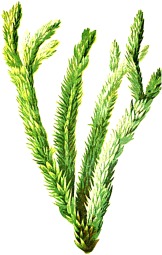
***Latin name***	*Digitalis* *purpurea* L.	*Legousia speculum-veneris* Fisch.	*Prunella* *vulgaris* L.	*Rumex conglomeratus* Murray	*Samolus* *valerandi* L.	*Scapania* *undulata* L.	*Betula* *pendula* Roth	*Galium* *odoratum* L.	*Huperzia* *selago* L.
**Common name**	Foxglove	Corn bellflower	Self-heal	Sharp dock	Water pimpernel	River startip	Birch	Woodruff	Fir clubmoss
**Welsh name**	Ffiol y ffud	Drycheigyauc	Wennela 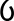 c, Ueidya 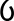 c	Turth, Twrch	Glaerllys	Gynglenyd	Uedl 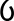 yn	Udrot	Thar 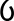 y mynyd
**Indication(s)**	abscess, heart	quinsy	carbuncle, toothache	pneumonia	Kidney stones	Abdominal, liver	Urinary	Pneumonia	Fertility
**Bioactive(s)**	digoxin 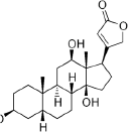	unknown	rosmarinic acid 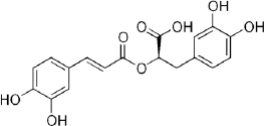	musizin 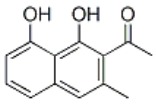	Unknown	Scapanol 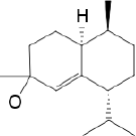	Betulin 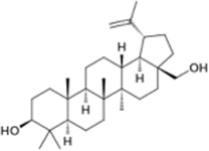	Asperulosidic acid 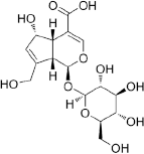	Huperzine A 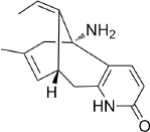

#### Foxglove, *Digitalis purpurea* L. (Plantaginaceae)—*Ffiol Y ffrud*

The plant is native to west and west-central Europe, including the British Isles. Foxglove is likely the closest claim to success for the Celtic and subsequent Welsh herbal tradition, as the plant was historically used to treat dropsy (congenital heart disease) and the decoction of its leaves was introduced into medical practice by the Scottish doctor William Withering in 1775. Chemical analysis revealed that foxglove contains a number of cardiac glycosides, including digoxin and digitoxin, that inhibit the activity of the Na/K-ATPase in the myocardium (Kjeldsen and Bundgaard, 2003). In the Digitalis Investigation Group clinical study (n = 6800), digoxin did not reduce overall mortality, but it reduced the rate of hospitalization both overall and for worsening heart failure (Digitalis Investigation Group, 1997). No CAM herbal supplements derived from foxglove are currently on the market due to safety and overdose concerns.

#### Corn Bellflower, *Legousia speculum-veneris* Fisch. (Campanulacaeae)—Dyrcheigyuac

Formerly known as *Campanula hybrida*, the plant is found in chalky cornfields of the British Isles and other parts of Europe. Due to its status as a weed, corn bellflower has been subjected to ecological and molecular phylogenetic studies, however there is no current scientific evidence of its medicinal properties, and its bioactive principles remain to be elucidated. *Myddfai I* cited it as a remedy for quinsy or tonsillar abscess.

#### Self-Heal, *Prunella vulgaris* L. (Lamiaceae)—Wennela
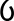
c, Ueidya
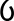
c

Although self-heal is common throughout Britain, Europe, Asia, and North America, it's traditional use was generally restricted to Asia, where it forms a part of traditional Chinese medicine targeting liver function, thyroid swellings, and inflammation. Flowering spikes of this plant contain up to 6% rosmarinic acid, one of the highest sources of this ingredient among plants (Lamaison et al., 1991). Otherwise, the phytochemical complexity of the plant is rather unknown. As a part of the polyherbal formulation, self-heal was explored as a pain reliver in combination with celecoxib in 181 Korean patients with rheumatoid arthritis (Song et al., 2007).

#### Sharp Dock, *Rumex conglomeratus* Murray (Polygonaceae)—Turth

Sharp dock is another plant of a wider Eurasian distribution that was utilized for its medicinal properties mostly outside of Western Europe. In Turkey, the plant locally known as *labada*, was used for treatment of purgative disorders and dysentery. The use of docks in Europe is predominantly restricted to the British Isles and the Carpathian basin (Vasas et al., 2015). The roots of docks are a rich source of anthraquinones and naphthalenes. *Myddfai I* recommends its use in treatment of pneumonia, which can be related to the antimicrobial properties of dock plants (Orbán-Gyapai et al., 2017). There is some certainty that the Welsh text is referring to *R. conglomeratus* in specific, due to the fact that species within the genus *Rumex* had unique Welsh names. Dock (*Rumex*) in general is often referred to as *tafolen.* Species follow such, *R. sanguineus* (*tafolen goch*), *R. crispus* (*tafolen grych*), *R. acutus* (*tafolen mair*), etc. The Welsh name *turth* appears to only have been attributed to *R. conglomeratus.*

#### Water Pimpernel, *Samolus valerandi* L. (Primulaceae)—*Glaerllys*

The plant is found in a variety of wet habitats all over Europe and other parts of the world. Due to a unique cosmopolitan distribution, water pimpernel has been subjected to extensive molecular phylogenetic studies (Jones et al., 2012), however there is no current scientific evidence of its medicinal properties, and its bioactive principles remain to be elucidated. The plant was also mentioned by Pliny in connection to Druid practices, providing another evidence for attribution of the water pimpernel to the Celtic and subsequent Welsh herbal tradition.

#### River Startip, *Scapania undulata* L. (Scapaniaceae)—*Gynglenyd*, *Cynglenydd*

Moss-looking leafy liverworts are the second largest group of plants, and their traditional use for addressing abdominal and liver disorders stemmed from a magical doctrine of signatures that believed in beneficial effects of plants to heal certain organs and body parts by resembling their shapes (Pearce, 2008). Traditionally, they were used crushed or intact to alleviate bruises, burns, and wounds, and their biochemistry exhibits a wide range of biologically active compounds (Asakawa, 2008). Liverworts, including *Scapania undulata*, contain highly specialized oil bodies enriched with sesqui- and diterpenoids, many of which have not been found in higher plants (Adio et al., 2004). *Scapania undulata* is the most common liverwort in South Wales and the name *cynglenydd* in the Welsh text is different than that of the generic *hepatica* (*redega
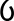
c*), suggesting a more detailed knowledge of this particular species. However, it is possible that this refers to the entire genus *Scapania.* This translation was corroborated by Davies, 1813.

### Spiritual Healing Practices

While the medicine of Greece and Hellenized Anatolia made great strides to separate formulations, vehicles, and single herb preparations (*simples*) into a pharmacologically-relevant treatments, *Myddfai I* herbal preserved the tradition of Roman and Celtic writers to incorporate magical formulas, scoring charms, evil spirits, and incantations into their healing practices. The reason could be argued that a later and often incomplete conversion to Christianity at the Celtic fringes of the British Isles preserved syncretism with pagan rituals including recognition of the fourfold principle, the luck of white cattle, eels, and roosters, lucky and unlucky days, as well as the legend of the Lady of the Lake—in which the authors described the way which they received healing powers—from what was essentially a water spirit.

Many herbal preparations also preserved the significance of numbers 3 and 9 in selection of ingredients, number of manipulations, and frequency of dosing—often a medicine made of three components was taken thrice daily for 9 days. The number 3 (and thus 9 = 3x3) was significant in Celtic religion prior to Christianity and in syncretic Celtic Christianity (Arthurian legends), symbolizing the trinity. However, the trinity itself predates Christianity in Indo-European religions including Roman, Celtic, Germanic, and Hindu polytheism and may be supported by the trifunctional hypothesis of Dumezil. Triple deities such as the Matronae (Gallic), the Morrigan (Irish), and Brigid (Irish), are prominent examples of the significance of three in Celtic culture along with symbols such as the triskele. In *Tain Bo Cuailnge,* a 12^th^ century Irish legend, the Morrigan appears in battle as three animals (an eel, a wolf, and a white cow), sustains three wounds, and is cured by three drinks of milk. Welsh mythology similarly abounds with the appearance of the number three, the deity Llyr has three children, and there are often three demons or plagues found in myths. The theme of threefold death is found in the Welsh *Myrddin Wyllt,* and this concept may be supported by the remains of the *c.* 1^st^ century AD Lindow bog body which sustained a hanging, head wound, and cut throat and was found with mistletoe pollen grains in his stomach (Hutton, 2011). Interestingly, Marcellus Empiricus references Esus in part of a remedy for treating throat infections, a Gallic god worshipped also in Roman Britain. Esus is part of the triple god entity containing Teutates and Taranis, documented by the Roman poet Lucan in the 1^st^ century AD, as well as depicted on the Pillar of the Boatman (along with a sacred bull, Tarvos Trigaranus).

A distinctive Celtic ethnographic framework of traditional healing beliefs and practices also survived in part with local population and a network of hereditary scholarly physicians of the Western Isles of Scotland (i.e. Macleans in Skye, O'Conachers in Argyll, Beatons in Islay and Mull) (Anonymous, 1906). A peculiar glimpse of these practices could be found in Martin Martin's *A description of the Western Islands of Scotland* (*ca.* 1695). Neil Beaton of Skye was said to treat “*Lilium Medicinæ, and some other practical pieces that he has heard of, with contempt*. *The success attending his cures was so extraordinary that people thought his performances to have proceeded rather from a compact with the devil, than from the virtue of simples. To obviate this, he pretends to have had some education from his father, though he died when he hurnself was but a boy.*” A more contemporary account of a direct oral transmission of herbal tradition of the Western Isles can be also found in the Maclagan Manuscripts (1892–1903) collected throughout the Highlands and the Inner Hebrides (Turner, 2014). More of this knowledge was also captured in the recent written records of Mary Beith (*Healing Threads*, 1995), Tess Darwin (The Scots Herbal, 1996), and Bridgewater & Milligan (Flora Celtica, 2004).

Contrary to magic formulas, charms, and incantations which may exert beneficial results on human body by affecting the neurobiological mechanisms of individual expectations and psychosocial placebo effects (Benedetti et al., 2005), herbal remedies fall within a category of evidence-based scientific research that can be applied to rigorously test the efficacy and safety of plant preparations, validate their traditional use, and provide novel biochemical leads to the existing drug development pipelines (Palatini and Komarnytsky, 2018). The herbal potential of medieval plant collections to fight microbial infections was successfully explored both for *Bald's Leechbook* (Harrison et al., 2015) and *Myddfai I* (Wagner et al., 2017), with the latter work also identifying additional plants effective against microbes from the modern tradition of the Scottish School of Herbal Medicine, including the leaves of the endemic Arran white beam (*Sorbus arranensis* Hedl.). These findings, however, only further strengthen the critical need for correct translations, botanical identification of vernacular and old language plant names, understanding changes in formulation, biochemical composition, and efficacy of medicinal plants that varies tremendously based on plant species, environmental location, and tissues used in the preparation.

## Conclusions

The Celtic fringe flora of the British Isles subsequent to the last glaciation shares a lot of common species with Scandinavian plants (those that likely survived glaciation in the southern or mountaintop refuges), and the incomers from the Germanic and Mediterranean flora that spread over the land bridges that existed at the time (Willis, 1995). As such, it is very difficult to differentiate between local versus continental herbal tradition that both share a very similar sets of plants. Westwards migrations of the Indo-European R1b tribes along the Danube and Rhine basins, superimposed on gathered plants automorphies found in the early settlements, clearly suggesting that the herbal knowledge of what later became Hellenized Anatolia was likely transferred to Central Europe and beyond, and this transfer coincided with the expansion of Celtic tribes. Appearance of some other plants of medicinal value was linked to later Roman introductions as in case with the greater celandine (*Chelidonium majus* L.) (Zielińska et al., 2018).

Traces of Celtic framework of traditional herbal remedies can still be found in classical and medieval herbal collections largely dominated by Mediterranean plants, as was shown for the foxglove (*Digitalis purpurea* L.) and several other plants. While the extent to which the later Welsh 14^th^ century text (*Myddfai I*) manifest an earlier oral tradition is uncertain due to overlaps with the classical writers, later scribes, additions, and even forgeries of the manuscript (*Myddfai II*), as well as dubious translations of plant identities and human diseases. Recipes from *Myddfai I*, however, carry distinct herbal preparation signatures (use frequencies of certain formulations and vehicles), which are vastly different from earlier Greco-Roman and contemporary Anglo-Saxon and Germanic traditions. The old practices also persisted in the Celtic fringes of the British Isles (Wales, Scotland, Ireland, and the Isle of Man) till very recently, but were rarely incorporated into the written tradition until the late 18–19^th^ centuries. Consequently, many herbal remedies remained an integral part of prevention and treatment of human diseases until modern times, some were rediscovered as botanical drugs and incorporated into pharmacopeias, while the other neglected recipes can be further explored for novel interactions and pharmacological applications.

## Author Contributions

CW translated Welsh text, identified plant names, health indications, and performed descriptive statistics of the herbal texts. SK developed the concept of herbal preparation signatures. CW, JD, and SK conceived, designed, and developed the framework of the study. CW and SK drafted and wrote the manuscript. JD edited the manuscript. All authors read and approved the manuscript.

## Conflict of Interest

The authors declare that the research was conducted in the absence of any commercial or financial relationships that could be construed as a potential conflict of interest.
