# Supplementary material for: Celtic Provenance in Traditional Herbal Medicine of Medieval Wales and Classical Antiquity
Source: Front Pharmacol. 2020 Feb 28;11:105. doi: 10.3389/fphar.2020.00105 (PMC7058801; doi:10.3389/fphar.2020.00105)
Supplement: Supplementary file 1 [file Table_1.docx]

**Supplementary Table 1. List of distinctive species gains or losses (autapomorphies) that defined the finds of wild gathered plants among the Neolithic settlements in Europe, with modifications after (Coward et al., 2008)**.

| **(1) Mediterranean coastal routes** | | | | | | |
| --- | --- | --- | --- | --- | --- | --- |
| **Southernmost**  **Greece** | **Sicily** | **Southern Italy** | **Portugal, Spain** | **Scotland** | **Ireland** | **Southern Britain** |
| Crete | Impressed ware, Stentinello | Impressed ware, Serra d'Alto, Diana, Lagnano da Piede | Impressed ware, Cardial, Chassey | Early | Early | Early |
| *+Rumex sanguineus* L. | *−Hordeum vulgare var. nudum* L. | *−­Vicia ervilia* L. | ***+Papaver somniferum* L.**  *+Pisum sativum* L. | *−Lens culinaris* Medik.  *−Triticum monococcum* L. | *−Hordeum vulgare var. nudum* L.  *−Triticum moncoccum* L. | *+Arrhenatherum elatius* L.  *−Asperula arvensis* L.  *−Chenopodium hybridum* L.  *−Echinochloa crus-galli* L.  *−Galium spurium* L.  *−Lens culinaris* Medik.  *−Pisum sativum* L.  *+Plantago major* L.  *+Poa trivialis* L.  *+Polygonum arenastrum* Boreau  *+Prunella vulgaris* L.  *−Rumex crispus* L.  *+Urtica urens* L. |
| **(2) Danube and Rhine valley routes** | | | | | | |
| **Thessalian Greece** | **Bulgaria Macedonia** | **Central Germany** | **Western Germany** | **Northern Italy** | **Austria, Western Carpathians** | **North France Benelux** |
| Sesklo | Karanovo, Starcevo | LBK | LBK | Square-mouthed pottery, Fagnigola, Impressed ware, Catignano | Eastern LBK, Koros | LBK, Swifterbant, Rubane, Group de Blicquy, Cerny |
| *+Agrostemma githago* L.  *+Ajuga chamaepitys* L.  *+Bromus secalinus* L.  *+Cicer arietinum* L.  *+Coriandrum sativum* L.  *+Galium aparine* L.  *+Galium spurium* L.  *+Linum usitatissimum* L.  *+Lolium temulentum* L.  ***+Panicum miliaceum* L.**  *+Portulaca oleracea* L.  ***+Verbena officinalis* L.** | *+Adonis flammea* Jacq.  ***+Agrimonia eupatoria* L.**  *+Ajuga chamaepitys* L.  *+Anagallis arvensis* L.  *+Aphanes arvensis* L.  ***+Atropa belladonna* L.**  *+Bromus arvensis* L.  *+Chenopodium murale* L.  *+Chenopodium polyspermum* L.  *+Cicer arietinum* L.  *+Convolvulus arvensis* L.  ***+Fragaria vesca* L.**  *+Galium mollugo* L.  *+Hibiscus trionum* L.  ***+Hyoscyamus niger* L.**  *+Lathyrus sativus* L.  ***+Plantago lanceolate* L.**  *+Poa annua* L.  *−Polygonum lapathifolium* L.  *+Polygonum minus* Huds.  *+Portulaca oleracea* L.  *+Rubus fruticosus* L.  *+Rumex crispus* L.  ***+Verbena officinalis* L.**  *+Veronica hederifolia* L.  *+Vicia ervilia* L.  *+Vicia tetrasperma* L. | ***+Agrimonia eupatoria* L.**  *+Asperula arvensis* L.  *+Bromus arvensis* L.  *+Chenopodium hybridum* L.  *+Euphorbia helioscopia* L.  ***+Hyoscyamus niger* L.**  ***+Panicum miliaceum* L.**  ***+Plantago lanceolate* L.**  *+Plantago major* L.  *+Polygonum aviculare* L.  *−Polygonum persicaria* L*.*  *+Rumex acetosella* L.  *+Vicia ervilia* L.  ***+Vicia faba* L.**  *+Vicia tetrasperma* L. | ***+Agrimonia eupatoria* L.**  *+Bromus arvensis* L.  *+Euphorbia helioscopia* L.  ***+Hyoscyamus niger* L.**  ***+Panicum miliaceum* L.**  ***+Plantago lanceolate* L.**  *+Plantago major* L.  *+Polygonum amphibium* L.  *−Polygonum persicaria* L.  *+Rumex acetosella* L.  *+Vicia ervilia* L.  ***+Vicia faba* L.**  *+Vicia tetrasperma* L. | *+Agrostemma githago* L.  ***+Fragaria vesca* L.**  *+Linum usitatissimum* L.  ***+Papaver somniferum* L.**  *+Polygonum convolvulus* L.  *+Polygonum persicaria* L.  *+Portulaca oleracea* L.  *+Rubus fruticosus* L. | *+Chenopodium album* L.  *+Galium tricornutum* Dandy  ***+Panicum miliaceum* L.**  *+Polygonum convolvulus* L. | *−Anagallis arvensis* L.  *−Arrhenatherum elatius* L.  *+Chenopodium ficifolium* Sm.  *−Papaver argemone* L.  *−Phleum pretense* L.  *−Poa trivialis* L.  *+Polygonum amphibium* L.  *−Polygonum arenastrum* Boreau  *−Prunella vulgaris* L.  *−Rubus fruticosus* L.  *−Veronica hederifolia* L. |
